# Supplementary figures and images for: Cells with Treg-specific FOXP3 demethylation but low CD25 are prevalent in autoimmunity
Source: J Autoimmun. 2017 Nov;84:75–86. doi: 10.1016/j.jaut.2017.07.009 (PMC5656572; doi:10.1016/j.jaut.2017.07.009)

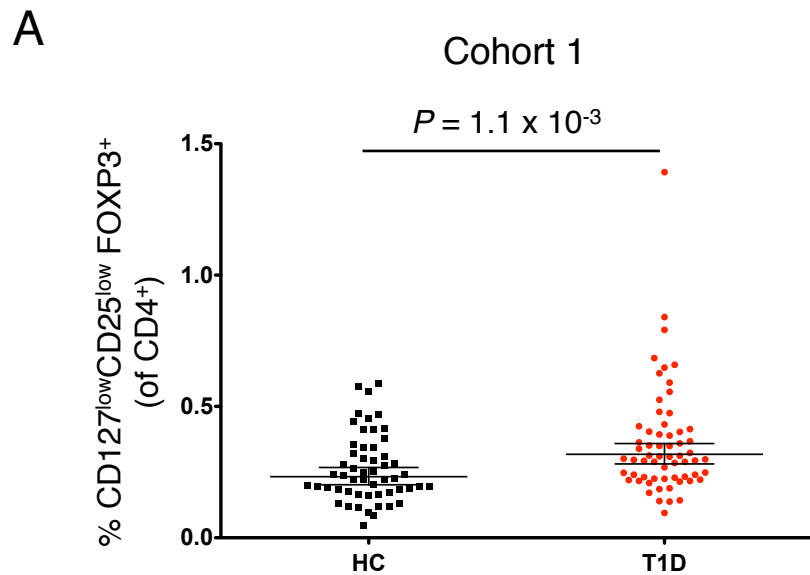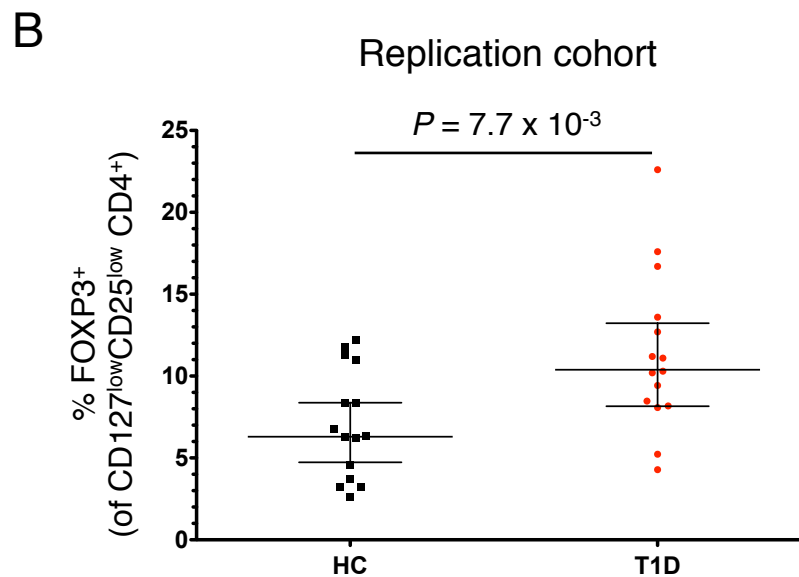

Supplement: Supplementary Fig. 1 — Frequency of CD127lowCD25lowFOXP3+T cells is increased in T1D patients. (A) Scatter plot depicts the total frequency (geometric mean ± 95% CI) of CD25lowFOXP3+ cells out of CD4+ T cells in our discovery cohort of 62 T1D patients (depicted by red circles) and 54 healthy controls (depicted by black squares) (B) Scatter plots depict the frequency (geometric mean ± 95% CI) of FOXP3+ cells from CD127lowCD25low T cells in: (i) an independent replication cohort consisting of 15 T1D patients and 15 healthy controls. P values were calculated using two-tailed unpaired t-tests comparing the geometric mean of the assessed immune subsets between T1D patients and healthy controls (HC). [file mmc1.pdf]

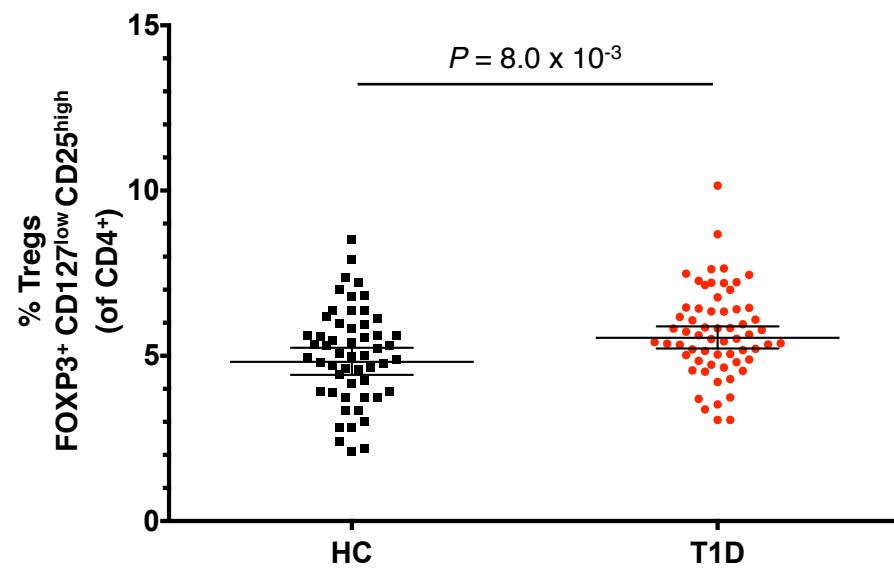

Supplement: Supplementary Fig. 2 — Minimal increase in the frequency of CD127lowCD25highFOXP3+T cells in T1D patients. Scatter plot depicts the total frequency (geometric mean ± 95% CI) of CD25highFOXP3+ cells (classical Tregs) out of CD4+ T cells in our discovery cohort of 62 T1D patients (depicted by red circles) and 54 healthy controls (depicted by black squares). P values were calculated using two-tailed unpaired t-tests comparing the geometric mean of CD25highFOXP3+ Tregs between T1D patients and healthy controls (HC). [file mmc2.pdf]

A

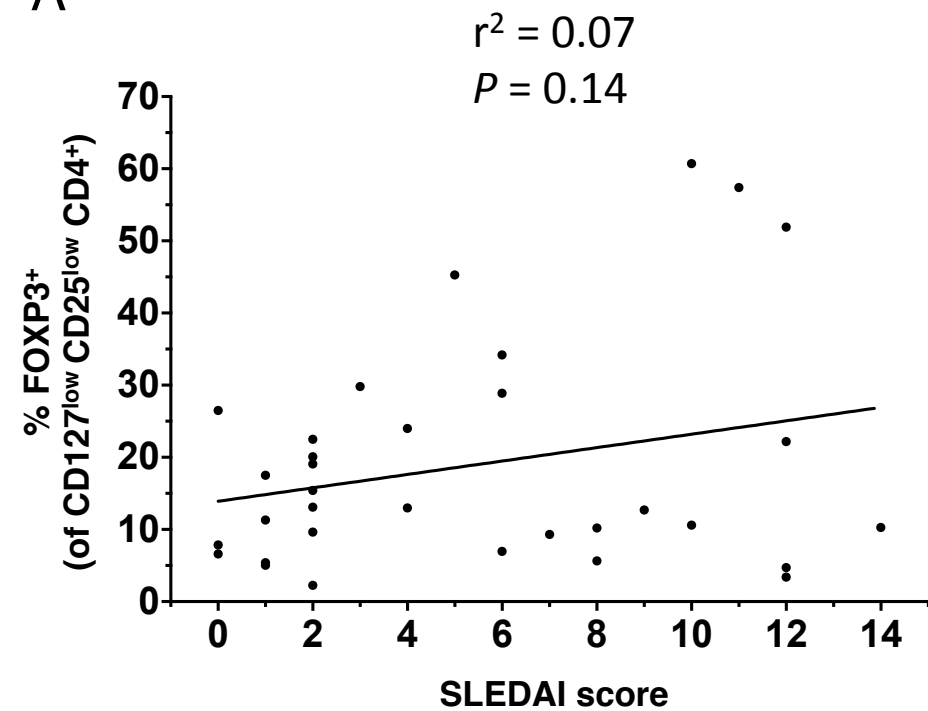

B

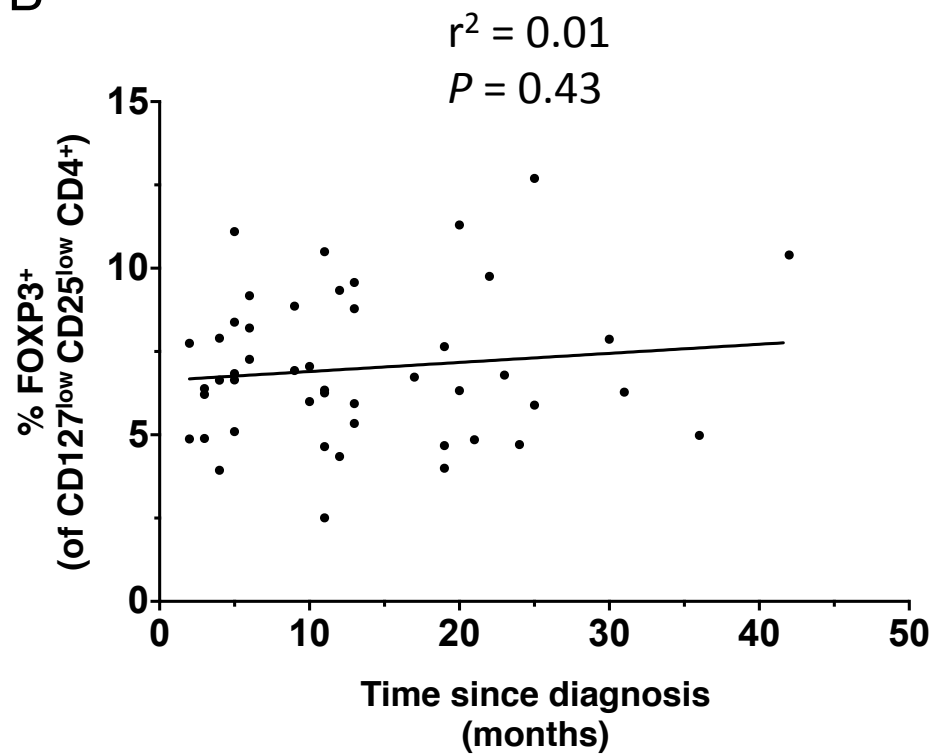

Supplement: Supplementary Fig. 3 — Association of the frequency of CD127lowCD25lowFOXP3+T cells with disease activity. (A) Data shown depicts the correlation between the frequency of FOXP3+ cells among CD127lowCD25low T cells and the SLE disease activity index (SLEDAI) at the time of sampling in SLE patients. (B) Scatter plot depicts the correlation between the frequency of FOXP3+ cells among CD127lowCD25low T cells and the time since diagnosis in 49 recently diagnosed T1D patients (median 11 months, range 2–42 months) from the D-GAP cohort. P values were obtained by linear regression analysis. [file mmc3.pdf]

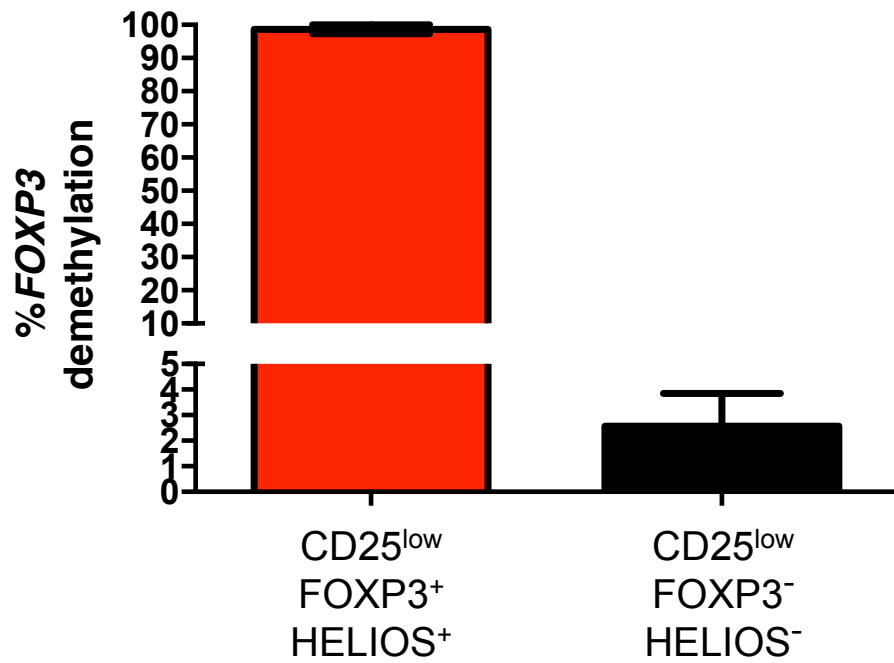

Supplement: Supplementary Fig. 4 — TSDR methylation profile of HELIOS+CD45RA−CD25lowFOXP3+cells is maintained in SLE patients. Frequency (mean ± SEM) of reads demethylated at eight or nine of the nine interrogated CpG sites in the FOXP3 TSDR in CD45RA− HELIOS+ CD25lowFOXP3+ cells and CD45RA− HELIOS− CD25lowFOXP3− Teffs. The data were obtained from sorted cells from three independent SLE donors. [file mmc4.pdf]

**A**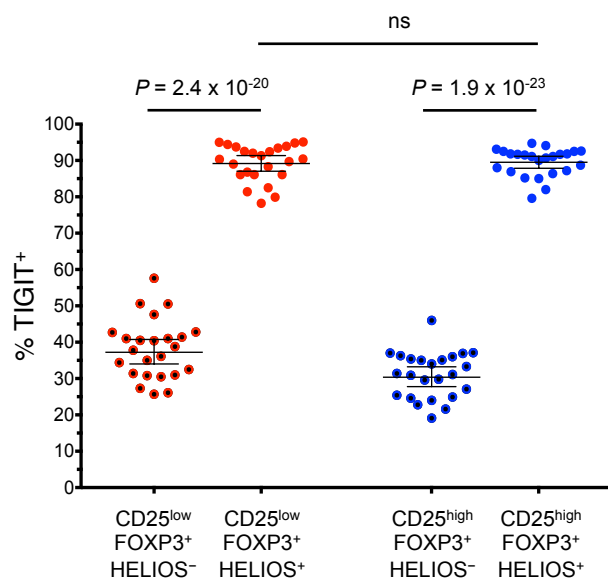**B**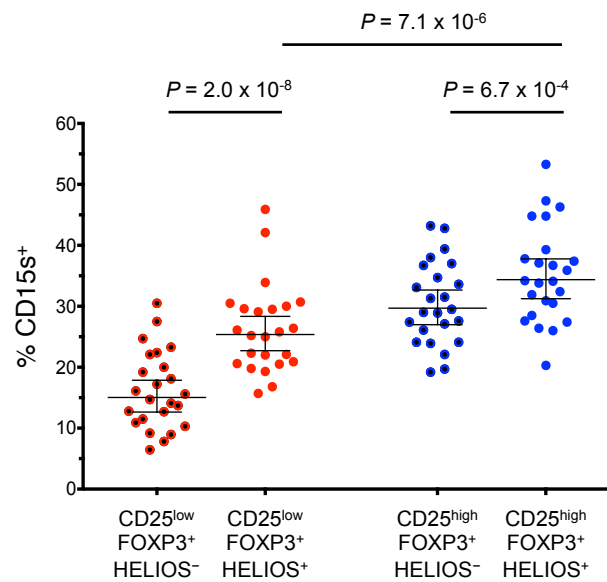**C**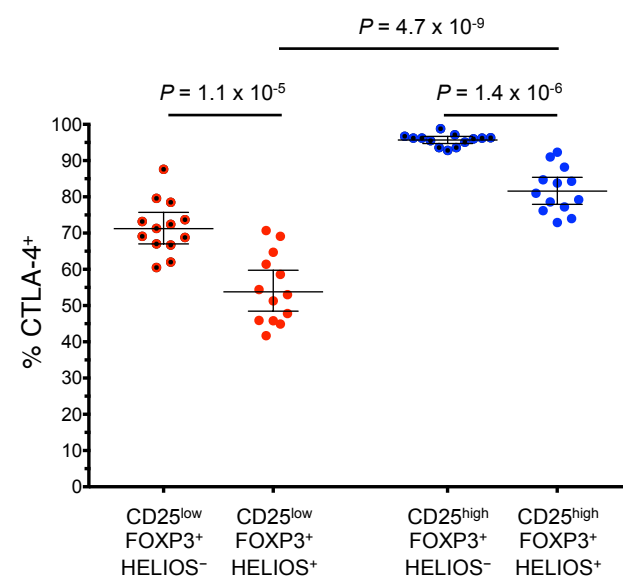**D**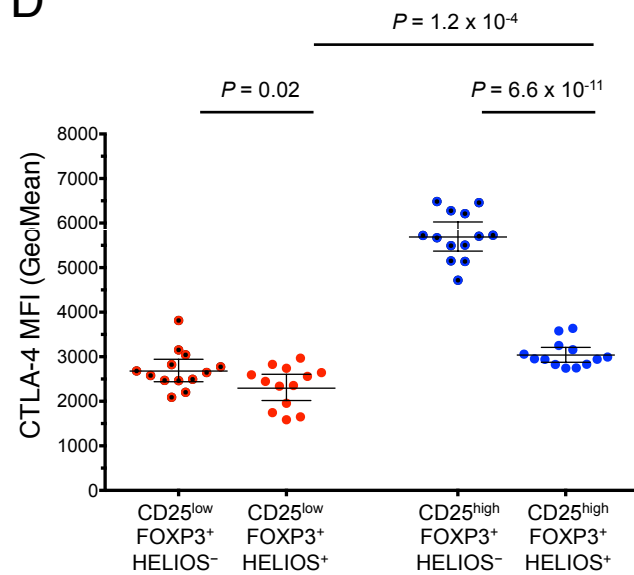**E**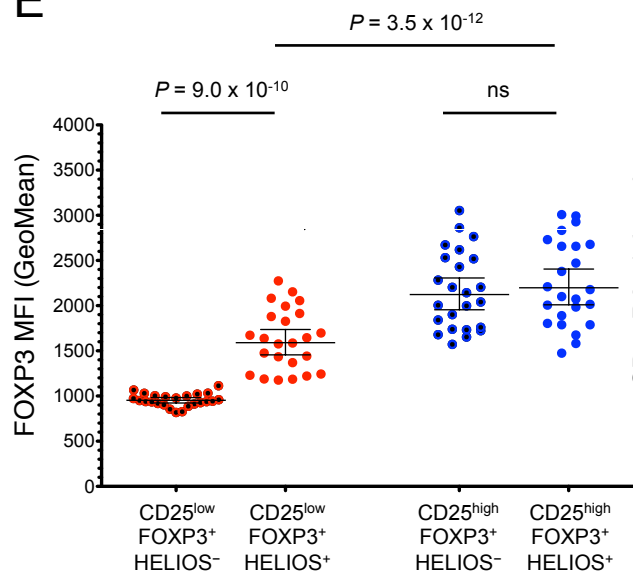**F**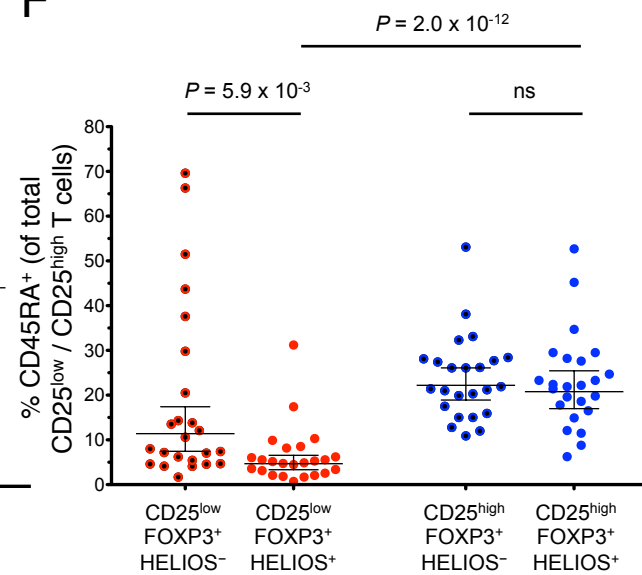

Supplement: Supplementary Fig. 5 — HELIOS expression defines distinct FOXP3+subsets. Scatter plots depict the distribution (geometric mean ± 95% CI) of TIGIT (n = 24) (A), CD15s (n = 24) (B), CTLA-4 (both frequency and MFI of the positive fraction; n = 13) (C, D), FOXP3 MFI (n = 24) (E) and CD45RA (n = 24) (F) in the HELIOS+ and HELIOS− fractions of the (i) CD25lowFOXP3+ T cells (depicted in red) and (ii) conventional CD25lowFOXP3+ Tregs (depicted in blue). P values were calculated using two-tailed paired t-tests. [file mmc5.pdf]

**A**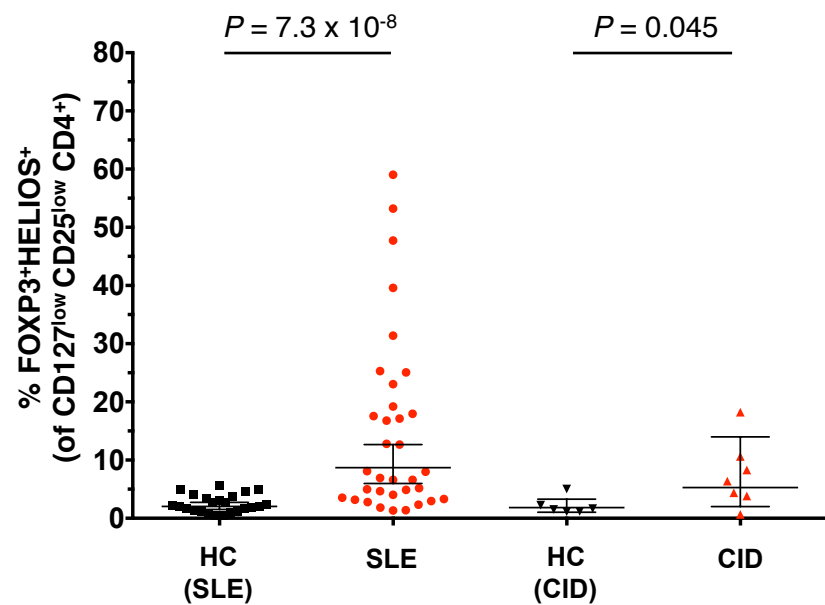**B**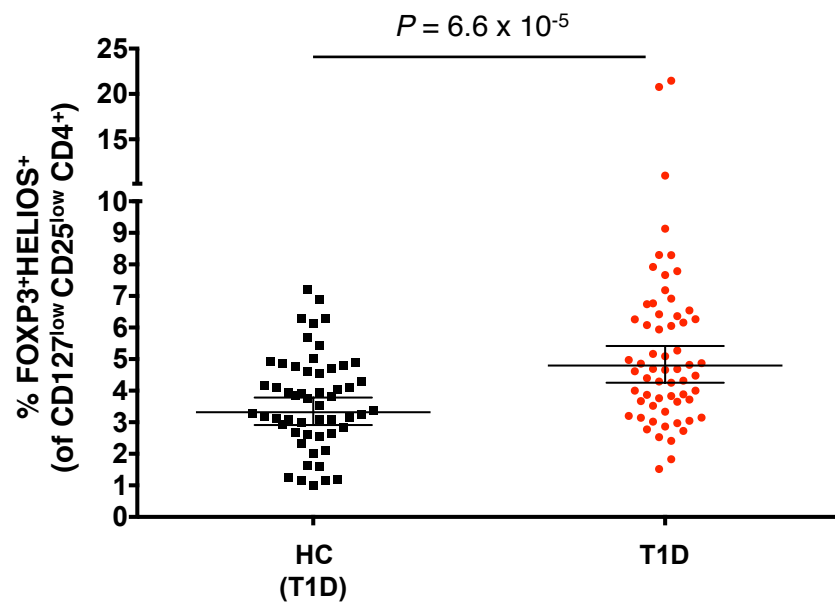**C**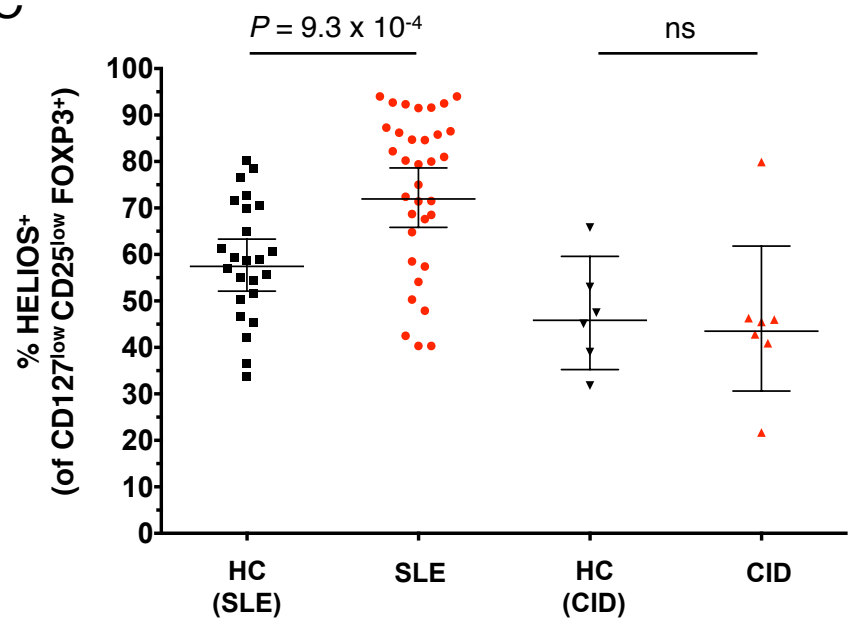**D**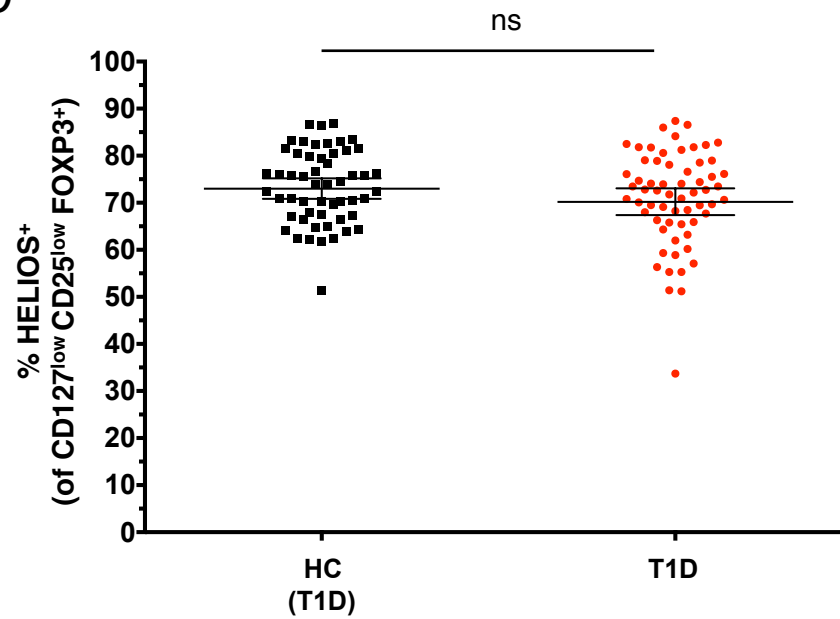

Supplement: Supplementary Fig. 6 — The frequency of HELIOS+CD25lowFOXP3+cells is increased in patients with autoimmune disease. (A, B) Scatter plots depict the distribution (geometric mean ± 95% CI) of HELIOS+FOXP3+ cells among CD127lowCD25low T cells in SLE patients (N = 34 patients vs 24 healthy donors) and combined immunodeficiency (CID) patients with active autoimmunity (N = 7 patients vs 6 healthy donors) (A); and in a cohort of T1D patients (N = 62; depicted by red circles) and healthy donors (N = 54; depicted by black squares) (B). (C, D) Scatter plots depict the distribution (geometric mean ± 95% CI) of HELIOS+ cells within CD25lowFOXP3+ T cells in the cohort of SLE and CID patients (C) and in the cohort of T1D patients (D). P values were calculated using two-tailed unpaired t-tests comparing the geometric mean of the assessed immune subsets between patients and the respective healthy control groups. .HC, healthy controls; T1D, type 1 diabetes patients; SLE, systemic lupus erythematosus patients; CID, combined immunodeficiency patients; ns = non-significant. [file mmc6.pdf]

A

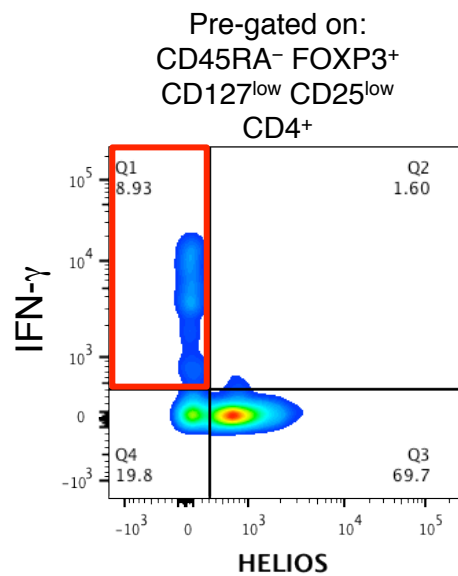

B

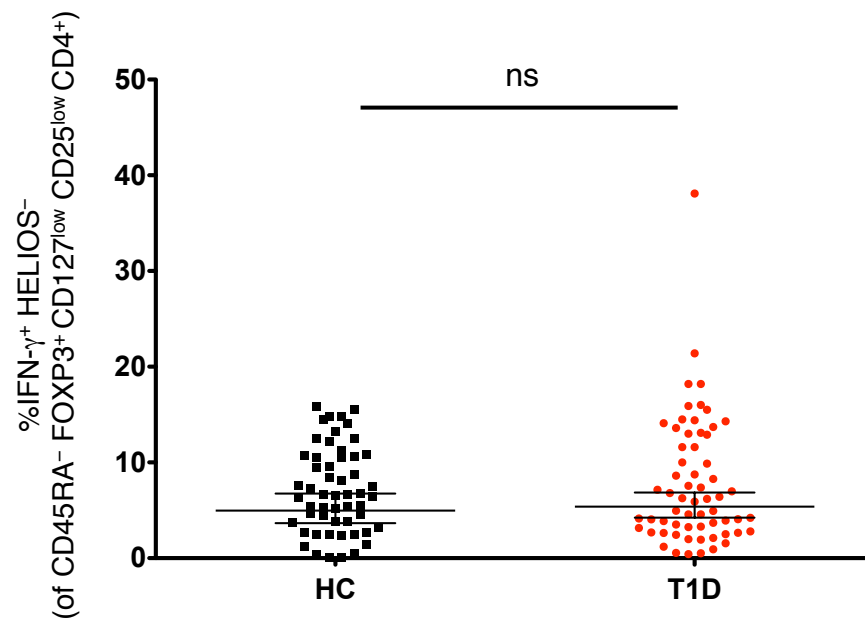

C

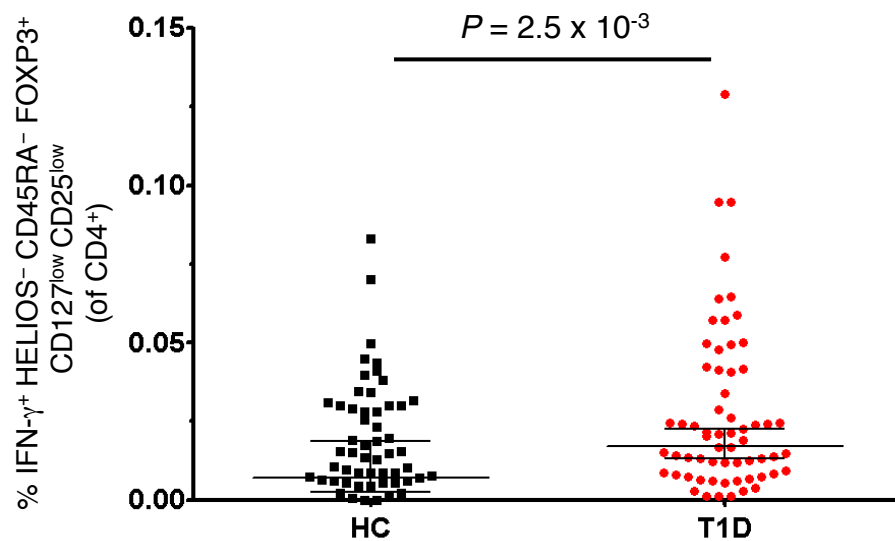

Supplement: Supplementary Fig. 7 — Production of IFN-γ from HELIOS−CD45RA−CD127lowCD25lowFOXP3+T cells is not altered in T1D patients. (A) Gating strategy illustrating the production of IFN-γ in the HELIOS− and HELIOS+ CD45RA− fractions of CD127lowCD25lowFOXP3+ cells. FACS gating plot is a representative example. (B) Plot depicts the distribution of the frequency (geometric mean ± 95% CI) of IFN-γ+ HELIOS− T cells in the CD45RA− CD127lowCD25lowFOXP3+ population. Frequency of IFN-γ+ cells was compared between T1D patients (N = 62; depicted by red circles) and healthy donors (N = 54; depicted by black squares) following in vitro stimulation with phorbol-12-myristate-13-acetate (PMA) and ionomycin. (C) Plot depicts the distribution of the frequency (geometric mean ± 95% CI) of IFN-γ+ HELIOS− T cells in the CD45RA− CD127lowCD25lowFOXP3+ population out of total CD4 T cells from the same donors as in (B). P values were calculated by linear regression of the log-transformed data, including batch as a covariate. HC, healthy controls; T1D, type 1 diabetic patients. [file mmc7.pdf]
